# Static Analysis of Wooden Beams Strengthened with FRCM-PBO Composite in Bending

**DOI:** 10.3390/ma16051870

**Published:** 2023-02-24

**Authors:** Piotr Kazimierz Sokołowski, Paweł Grzegorz Kossakowski

**Affiliations:** Faculty of Civil Engineering and Architecture, Kielce University of Technology, Al. Tysiąclecia Państwa Polskiego 7, 25-314 Kielce, Poland

**Keywords:** laboratory tests, timber, timber structures, composites, FRCM, PBO, timber reinforcement

## Abstract

The article presents an analysis of the static work of bent solid-wood beams reinforced with FRCM–PBO (fiber-reinforced cementitious matrix–p-phenylene benzobis oxazole) composite. In order to ensure better adhesion of the FRCM–PBO composite to the wooden beam, a layer of mineral resin and quartz sand was applied between the composite and the wooden beam. Ten wooden pine beams with dimensions of 80 × 80 × 1600 mm were used for the tests. Five wooden beams, unreinforced, were used as referenced elements and another five were reinforced with FRCM–PBO composite. The tested samples were subjected to a four-point bending test in which the static scheme of a simply supported beam subjected to two symmetrical concentrated forces was used. The main purpose of the experiment was to estimate the load capacity, the flexural modulus and the maximum bending stress. The time needed to destroy the element and the deflection were also measured. The tests were carried out based on the PN-EN 408: 2010 + A1 standard. The material used for the study was also characterized. The methodology and assumptions adopted in the study were presented. The tests confirmed a significant increase in destructive force by 141.46%, maximum bending stress by 118.9%, modulus of elasticity by 18.32%, time needed to destroy the sample by 106.56% and deflection by 115.58% compared to the reference beams. The unusual method of wood reinforcement presented in the article can be considered as innovative, characterized not only by a significant load capacity margin exceeding 141%, but also by simplicity of application.

## 1. Introduction

Wooden structures have been known and used by man for millennia. Wood can perform structural functions no worse than steel or concrete provided that it is properly protected and maintained. Unfortunately, over the years, it requires repair or strengthening among others due to atmospheric, chemical, biological factors, temperature changes, destructive environmental effects, design and implementation errors and also when the load on a given element increases, which forces it to increase its load capacity and forces it to take appropriate measures actions aimed at obtaining a higher bearing capacity.

Currently, traditional and proven methods of strengthening wooden structures are willingly replaced by modern systems based on synthetic polymers [1,2,3,4,5,6,7,8,9,10,11,12,13,14,15]. Composites—because we are talking about them; they were created as a result of the search for materials that would be characterized by better strength, physical and chemical parameters than traditional materials with greater strength stiffness and possibly low density. As for this group, it is one of the most interesting achievements of materials science.

Reinforcement of structural elements with the use of FRP (fiber-reinforced polymers/plastics) composite materials, resulting from the combination of at least two different components at the macroscopic level is a relatively new and still developing issue. It was not until the end of the 1960s that epoxy resins with high strength properties were obtained, which resulted in attempts to use them in building structures [16]. Unfortunately, the mechanical properties of the FRP system depend mainly on the temperature and, in particular, on the glass transition temperature of the epoxy resin, which determines the durability and effectiveness of the reinforcement regardless of the fibers used. After exceeding the glass transition temperature, the matrix loses its properties and the FRP system becomes an ineffective reinforcement.

In response to this problem, the FRCM (fiber-reinforced cementitious matrix) system was developed for reinforcing reinforced concrete, concrete and masonry structures [17]; however, so far, it has not been tested in wooden structures. In the FRCM system, the resin matrix has been replaced with an inorganic mineral mortar, which covers the reinforced element and which connects to the fibers of the composite mesh, ensuring its adhesion to the reinforcement. Most often, one of the reinforcing elements is a PBO fiber mesh (p-phenylene benzobis oxazole). FRCM is an alternative to the FRP system mainly due to the mechanical properties of the system, not dependent on its temperature. In FRP systems, the composite is connected to the reinforcing element with an epoxy resin. the glass transition temperature of which is in the range of 40–80 °C. It is the glass transition temperature of the resin that determines the durability and effectiveness of the reinforcement, regardless of the fibers used. After exceeding the glass transition temperature, the matrix loses its properties and the FRP system loses its adhesion between the resin and the fibers and becomes an ineffective reinforcement. The FRCM system corrects this problem and, therefore, can be used in environments exposed to high temperatures or fire. According to the literature [18,19,20,21], tests carried out on bending beams reinforced with PBO–FRCM have shown that this system allows for effective reinforcement; however, the load capacity increase is lower than in the case of FRP reinforcements. This is due to the slip effect that occurs between the mineral mortar and the fibers. The reason is the mineral mortar, which is not able to cover all the fibers as thoroughly as epoxy resin. The destruction occurs as a result of premature detachment of the fibers from the matrix. This leads to incomplete use of the mechanical properties of PBO fibers. To increase the effectiveness of FRCM reinforcements, additional PBO mesh anchors should be used, which can prevent premature detachment of fibers and, thus, increase the use of the PBO mesh load capacity.

Composite materials are recommended for reinforcing almost any type of structure, such as: reinforced concrete, masonry, wooden, rarely steel. Load-bearing elements, such as ceilings, joists, columns and beams, are most often reinforced. The main reason for the reinforcement is to obtain a higher load capacity and stiffness of the element with the lowest possible density of the used reinforcement material. In the case of bent elements, reinforcement is most often used in the lower fibers subjected to stretching. However, the decision on the method of reinforcement should be preceded by a thorough assessment of the technical condition of the structure or its element.

The basic parameters describing the work of the element during bending, in addition to determining the destructive force, is the modulus of elasticity in bending (Young’s modulus) together with the maximum bending stress. Young’s modulus is a proportionality factor between a unit strain and the stress causing it. When estimating the modulus of elasticity, the range in which the strains occurring in both the compressed and stretched fibers of the cross-section do not exceed the elastic strains is analyzed, and the deflection arrow of the element is the measure of these strains [22,23]. In accordance with PN-EN 408: 2010 + A1 [24], this range has been normalized within the limit of 10% to 40% of the element’s destructive force.

In this study, the static work of pine wood reinforced with the FRCM–PBO composite was analyzed. Section 2 describes the methodology, assumptions adopted in the tests, how to determine the maximum force, maximum bending stress, section modulus of bending strength and stiffness modulus. The research stand and the course of research were described. The material used for the tests was also characterized, along with the basic physical properties of the tested elements of the A and F series. Section 3 presents results and discussion. Section 4 contains conclusions from the conducted experimental research.

## 2. Description of the Research

### 2.1. Methodology: Assumptions Adopted in the Research

The subject of the research was the use of the FRCM–PBO composite to reinforce bent solid wooden beams made of pine. Ten pieces of beams with dimensions of 80 × 80 × 1600 mm were used in the tests, five wooden beams were used as reference—unreinforced (series A) and the next five were reinforced with FRCM–PBO composite (series F). The main objective of the experiment was to estimate the effectiveness of the reinforcement by estimating the load capacity, the modulus of elasticity in bending and the maximum bending stress, as well as the time needed to failure of the element and the deflection.

**Figure 1:**
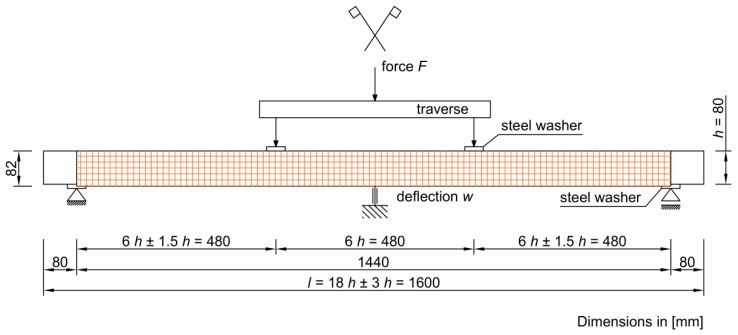
Scheme and static diagram of tested elements.

The estimation of the general modulus of elasticity consisted in carrying out a four-point bending test described in the PN-EN 408: 2010 + A1 standard [24]. A static diagram of a simply supported beam loaded symmetrically with two concentrated forces was used (Figure 1). In order to minimize local dents. a steel washer was used between the thrusts and the tested element. The applied forces were applied at a distance equal to 6 times the height of the cross-section from the axis of the supports to the upper surface of the element. During the tests. the thrusts moved at a constant speed specified in the standard [24]:

v = 0.003∙h [mm/s](1)

where:

h—height of the test sample [mm].
One level of load velocity v = 0.3 mm/s was assumed—for beams of the A and F series.

The bending modulus was determined according to the formula contained in the standard [24]:

(2) $${\;E}_{m,g}=\frac{3{\mathrm{al}}^{2}-4a^{3}}{2{\mathrm{bh}}^{3}\left(2\;\frac{w_{2}-{\;w}_{1\;}}{F_{2}-{\;F}_{1}}-\frac{6_{a}}{5\mathrm{Gbh}}\right)}$$

wherein:

E_m,g_—modulus of elasticity [GPa];
B—width of the cross-section [mm];
H—height of the cross-section [mm];
L—span of the tested element in the axes of supports [mm];
F_2_ − F_1_—load increase in the linear range [N];
w_2_ − w_1_—deflection increase corresponding to the load increase [mm];
a—distance from the point of application of the concentrated force to the nearest support according to Figure 1 [mm].

The maximum force F_max_ was determined according to the recommendations of the standard [24] where the breaking load was applied at a constant speed not greater than ± 0.003 h (mm/s) and was v = 3 mm/s. The maximum load applied did not exceed 0.4 F_max_.

**Figure 2:**
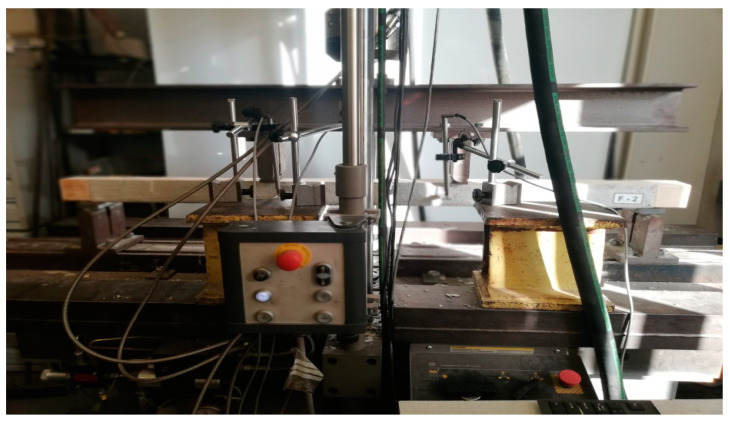
View of the test stand.

The maximum bending stress σ_m_ was calculated from the formula:

(3) $$\sigma_{m}=\frac{{\mathrm{aF}}_{\max}}{2W}$$

wherein:

σ_m_—maximum bending stress [MPa];
F_max_—maximum destructive force [N];
a—distance from the point of application of the concentrated force to the nearest support [mm];
  W—bending section modulus;

The bending section modulus W was calculated from the formula:

(4) $$W=\frac{{\mathrm{bh}}^{2}}{6}$$

wherein:

W—bending section modulus [mm];
b—sample width [mm];
h—sample height [mm];

The test stand included an MTS-type hydraulic testing machine (Figure 2), inductive sensors and software recording the tested parameters. During the tests, the loading force (F_max_), the deflection in the middle of the span (w) and the time from the beginning to the moment of element failure (t) were recorded continuously. The measurement of the deflection was made at the level of the lower extreme fibers compressed in the middle of the span of the element. After the tests. the density and humidity of the elements were measured using the WRD-100 hygrometer according to the PN-EN 384:2004 standard [25].

### 2.2. Material

**Figure 3:**
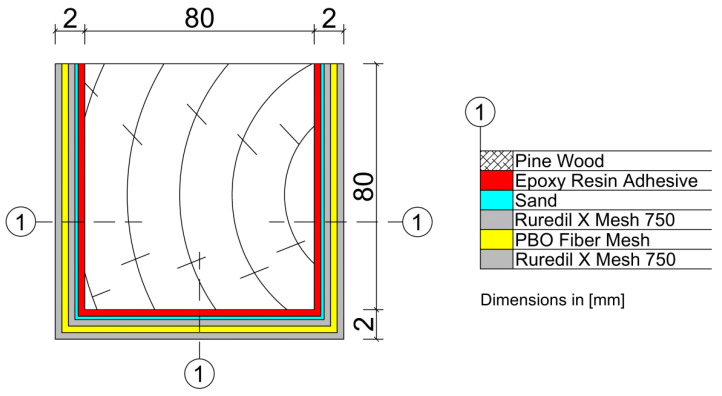
Cross-section of the F-series beams strengthened by FRCM–PBO system.

Ten pieces of wooden beams made of pine with dimensions of 80 × 80 × 1600 mm were used for the tests. Five wooden beams were used as referenced elements—unreinforced (marked A). The next ones were reinforced with FRCM–PBO composite (marked F) as shown in Figure 3. The average moisture content of the beams for A series was 13.75% and for F series 13.68%. The average density of the beams for A series was 0.000467 g/mm^3^ and for F series 0.000606 g/mm^3^. As mentioned at the beginning, the FRCM–PBO system was created to strengthen the reinforced concrete, concrete and masonry structures. However, its strength parameters suggest that it can be used to strengthen wooden structures due to its low weight, which increased on average compared to the A series by only 1900 g. In addition, the system offers quick and easy assembly increasing the flexural strength of structural elements, while having a positive effect on formability. It owes its unique properties to the combination of a mesh made of PBO fibers and a mineral matrix, which serves as a binder that transfers stresses and deformations of the structure to the fibers. The mechanical properties of the PBO fiber enable a significant increase in the load capacity of structural elements. PBO fibers are a material with high mechanical strength, where the tensile strength is 5.8 GPa compared to 4.1 GPa for carbon fibers that are sunk. The above-mentioned chemical bond ensures the adhesion of both components of the FRCM system; i.e., PBO fibers and the mineral matrix ensuring durability and excellent properties of the resulting composite. In order to ensure better adhesion of the FRCM–PBO composite to the wooden beam, a layer of mineral resin and a thin layer of resin sand was applied between the composite layer and the wooden beam. The applied roughening significantly improved the adhesion of the mineral mortar to the surface of the wooden beam, which was described in detail in the literature [26].

The use of FRCM–PBO for strengthening wooden elements makes the research original and at the same time makes it a useful solution for strengthening wooden structures, which has been extensively proven in the literature [27]. Unfortunately, at the moment, there are no examples of practical use of the composite in reinforcing wooden structures.

The method of strengthening FRCM–PBO can be considered innovative and, in particular, innovative for the 21st century due to the non-flammable properties of the mineral matrix and PBO fibers. Nevertheless, this issue requires a more extensive analysis and further experimental research.

The tests were carried out on elements on a technical scale due to the limited amount of material to be analyzed (wood, PBO mesh, mineral mortar), which allowed for the preparation of 10 samples and the technical capabilities of the MTS hydraulic testing machine. Therefore, an extensive statistical analysis was carried out detailing the effect of reinforcement on the improvement of strength characteristics manifested in the form of increasing destructive forces, stiffness and modulus of elasticity, which is described in detail in the literature [27].

The standard [24] defines the dimensions of the samples to be tested, while the technical capabilities of the MTS testing machine impose the use of materials on a reduced scale and length not exceeding 1600 mm.

The basic physical properties of the elements used for the tests for series A and F are presented in Table 1 and Table 2.

## 3. Results and Discussion

**Figure 4:**
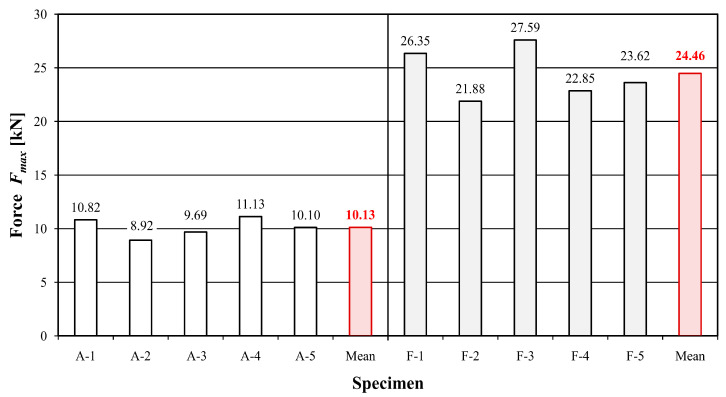
Values of destructive forces F_max_ of the tested beams.

**Figure 5:**
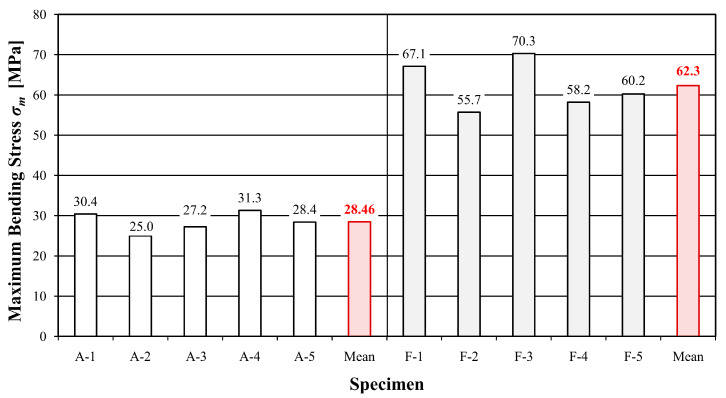
Values of the maximum bending stress σ_m_.

**Figure 6:**
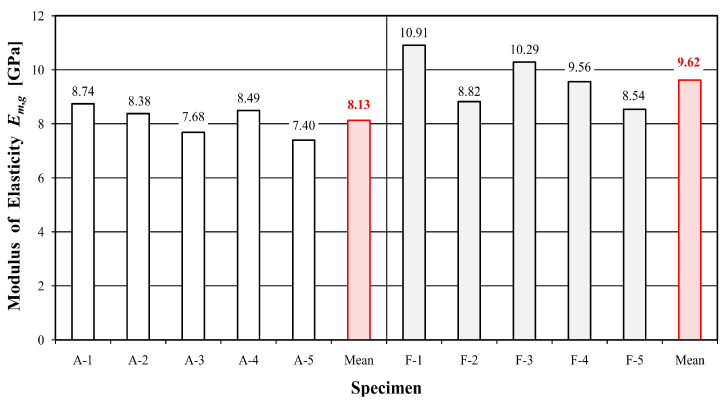
Values of modulus of elasticity E_m,g_.

Table 3 and Table 4 show the values of time, deflection, destructive force, maximum bending stress and modulus of elasticity for individual samples of unreinforced and reinforced beams, while Figure 4, Figure 5 and Figure 6 present a graphical illustration of the obtained results.

Beams reinforced with the FRCM–PBO composite were characterized by the longest test time of 106.56% compared to the reference beams. A significant increase in load capacity, maximum bending stress and deflection was also obtained, with values of 141.46, 118.9 and 115.58%, respectively. An increase in the stiffness modulus was also proven, although to a slightly lesser extent than originally expected, which amounted to 18.32%. The reason is the increase in deflection and the low stiffness of the PBO mesh. Nevertheless, the observed increase in deflection is also positive due to the extension of the time needed for failure and the behavior of the load-bearing element for a longer time during a possible overload.

**Figure 7:**
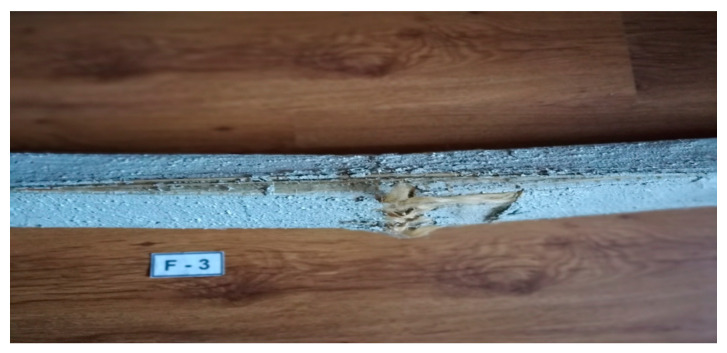
Example of beam failure caused by slippage of the FRCM–PBO composite.

Destruction of the beams in the mineral matrix occurred as a result of the occurrence of the sliding effect between the mortar and the fibers characteristic for this FRCM–PBO composite, i.e., premature detachment of the matrix from the beam. This was manifested by sudden cracks in the beams in the most weakened places—knots. The damage was visible in the lower and side fibers of the beams as shown in Figure 7; however, the increase in the tested parameters was greater than in the case of the reference beams. This manifested itself in the form of a sudden breakage without any signaling of the following failure. The beams cracked in a place weakened by the occurrence of a natural wood defect—knots in the lower fibers and partially in the side ones.

The load speed of 0.3 mm/s was identical for the A and F series beams and was determined in accordance with the requirements of the standard [24].

From a practical point of view, the presented method of reinforcement is characterized not only by good strength parameters, but also by low own weight, speed of execution, good thermal properties of the mineral matrix PBO fibers and corrosion resistance. There is no need for wood impregnation. At the moment, there are no examples of practical use of the FRCM–PBO composite for reinforcing wooden structures and, thus, no assumptions that could limit its practical use.

**Figure 8:**
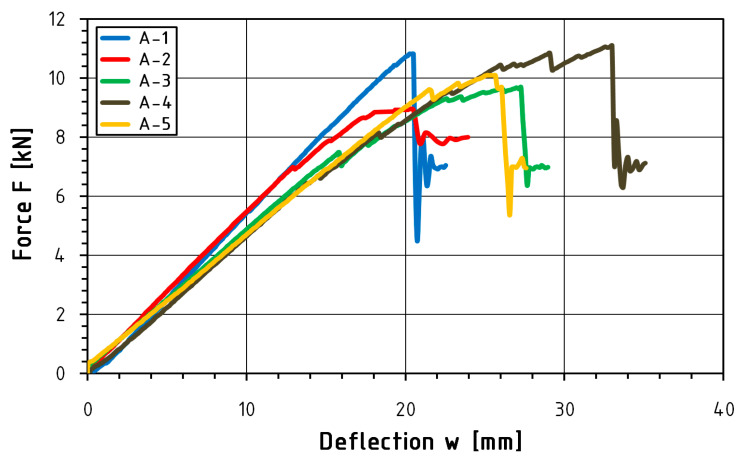
Force-deflection equilibrium path for A-series beams.

**Figure 9:**
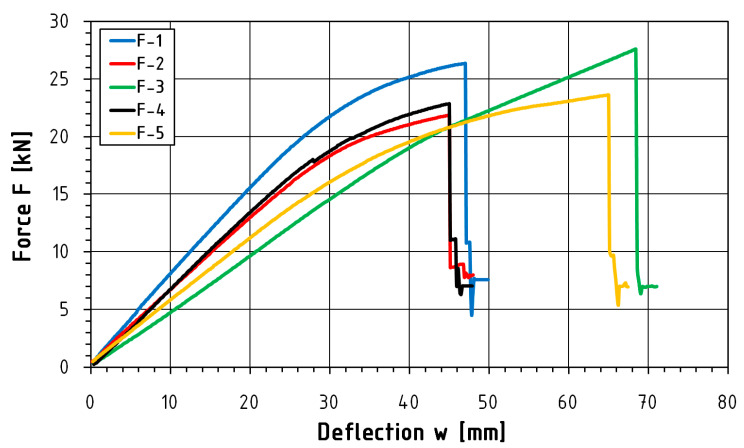
Force-deflection equilibrium path for F-series beams.

This study presents a load-deflection graph (Figure 8 and Figure 9) for the entire course of the test until failure. Statistical analysis was carried out for the range from 0.1 F_max_ to 0.4 F_max_. Deflection measurements carried out using inductive sensors measured at the level of the lower fibers compressed in the middle of the beam span are characterized by abrupt changes in value. Such changes in course are influenced by the sudden rapture of the element resulting from the achievement of tensile strength in the case of reinforced elements. Table 5 and Table 6 present a summary of the statistical analysis of the results of static tests of beams from the A and F series, while Table 7 illustrates a comparison of the test results of both series.

## 4. Conclusions

The article presents an analysis of the static work of bent pine wooden beams reinforced with the FRCM–PBO composite on a technical scale with dimensions of 80 × 80 × 1600 mm in accordance with the PN-EN 408: 2010 + A1 standard [24].

Based on the analysis of the results of four-point bending tests, comparing the average values of the obtained results, a significant increase was observed:

− destructive force by 141.46%;
− maximum bending stress by 118.9%;
− modulus of elasticity by 18.32%;
− time needed to destroy the sample by 106.56%;
− deflections by 115.58%.

Additionally observed:

− no influence of the load speed on the change of strength parameters;
− slip-effect characteristic of the FRCM–PBO composite occurring between the mortar and the fibers, manifested by premature detachment of the fibers from the matrix.

Therefore, it can be concluded that the FRCM–PBO composite is an alternative to FRP systems for strengthening wooden structures and, thus, a beneficial solution for reinforcing beams and other wooden bending elements. Nevertheless, the problems of this type of reinforcement due to the complexity of the mechanical and physical properties of wood and the chemical structure of the composite require a more extensive analysis and further experimental research.

## Figures and Tables

**Table 1:** Basic physical properties of the tested elements of the A series.

Beam Number	Dimensions (mm)	Density (g/mm^3^)	Humidity (%)	Beam Weight without Reinforcement (g)	Mass of Finishing Layers and Reinforcements (g)	Total Mass of the Beam (g)
A-1	80 × 80 × 1600	0.000467	13.97	4777	-	4777
A-2	80 × 80 × 1600	0.000465	13.60	4762	-	4762
A-3	80 × 80 × 1600	0.000465	13.35	4766	-	4766
A-4	80 × 80 × 1600	0.000468	14.25	4788	-	4788
A-5	80 × 80 × 1600	0.000468	13.62	4793	-	4793

**Table 2:** Basic physical properties of the tested elements of the F series.

Beam Number	Dimensions (mm)	Density (g/mm^3^)	Humidity (%)	Beam Weight without Reinforcement (g)	Mass of Finishing Layers and Reinforcements (g)	Total Mass of the Beam (g)
F-1	84 × 82 × 1600	0.000603	14.25	4795	1848	6643
F-2	84 × 82 × 1600	0.000600	13.30	4789	1820	6609
F-3	84 × 82 × 1600	0.000609	13.57	4785	1925	6710
F-4	84 × 82 × 1600	0.000613	13.32	4791	1967	6758
F-5	84 × 82 × 1600	0.000605	13.95	4786	1881	6667

**Table 3:** Results of the tests of A-series beams.

Beam Number	Time t (sek)	Deflection w (mm)	Destructive Force F_max_ (kN)	Modulus of Elasticity E_m,g_ (GPa)	Maximum Bending Stress σ_m_ (MPa)
A-1	159.2	20.43	10.82	8.74	30.4
A-2	149.0	19.50	8.92	8.38	25.0
A-3	205.0	27.09	9.69	7.68	27.2
A-4	232.2	33.02	11.13	8.49	31.3
A-5	191.8	25.43	10.10	7.40	28.4

**Table 4:** Results of the tests of F-series beams.

Beam Number	Time t (sek)	Deflection w (mm)	Destructive Force F_max_ (kN)	Modulus of Elasticity E_m,g_ (GPa)	Maximum Bending Stress σ_m_ (MPa)
F-1	342.8	46.66	26.35	10.91	67.1
F-2	329.4	44.87	21.88	8.82	55.7
F-3	507.2	68.77	27.59	10.29	70.3
F-4	329.8	44.92	22.85	9.56	58.2
F-5	426.2	65.23	23.62	8.54	60.2

**Table 5:** Statistical analysis of the results of static tests of A-series beams.

	Time t (sek)	Deflection w (mm)	Destructive Force F_max_ (kN)	Modulus of Elasticity E_m,g_ (GPa)	Maximum Bending Stresses σ_m_ (MPa)
Arithmetic average	187.4	25.09	10.13	8.13	28.46
Standard deviation	33.9	5.47	0.88	0.56	2.51
Coefficient of variation	0.18	0.21	0.08	0.07	0.08

**Table 6:** Statistical analysis of the results of static tests of F-series beams.

	Time t (sek)	Deflection w (mm)	Destructive Force F_max_ (kN)	Modulus of Elasticity E_m,g_ (GPa)	Maximum Bending Stresses σ_m_ (MPa)
Arithmetic average	387.1	54.09	24.46	9.62	62.3
Standard deviation	78.30	11.87	2.41	0.99	6.16
Coefficient of variation	0.20	0.21	0.09	0.1	0.09

**Table 7:** Comparison of test results of A and F series.

Parameter	Series A	Series F	Increase (%)
Destructive force (kN)	10.13	24.46	141.46
Modulus of elasticity (GPa)	8.13	9.62	18.32
Maximum bending stresses (MPa)	28.46	62.30	118.9
Time (sek)	187.40	387.10	106.56
Deflection (mm)	25.09	54.09	115.58

## Data Availability

Not applicable.
